# From suffering towards communal well-being: experiences of a Dalcroze-inspired workshop for a community in crisis

**DOI:** 10.3389/fpsyg.2023.1176691

**Published:** 2023-08-04

**Authors:** Liesl van der Merwe, Debra Joubert, Johann Wilhelm Tempelhoff

**Affiliations:** ^1^School of Music & MASARA, North-West University, Potchefstroom, South Africa; ^2^Social Transformation, North-West University, Vanderbijlpark, South Africa

**Keywords:** Dalcroze, embodiment, musicking, Parys, social interaction, conflict resolution, health and well-being, community music

## Abstract

This qualitative hermeneutic phenomenological study aims to describe the meaning that members of the Parys community ascribed to their experiences of a Dalcroze-inspired workshop. Stakeholders were a number of local residents of Parys, a scenic town situated on the banks of the Vaal River in South Africa’s Free State Province. Local residents were asked to share their water-related experiences at a workshop organized by a North-West University research group. It became clear from their stories that they had experienced severe stressful circumstances. They had suffered stress because of the health risks posed by polluted water and the frequent lapses in the town’s water supply system. Dalcroze-inspired activities were used to facilitate this meeting. Data were collected through focus group interviews, open-ended individual interviews, photos, videos, and observations. All these data were consolidated in one heuristic unit in ATLAS.ti, a computer-assisted qualitative data analysis software program. The codes were organized into categories and themes. Friese’s (2014) notice, collect and think (NCT) method for computer-assisted qualitative data analysis was used. From the data analysis, five themes emerged related to the Dalcroze-inspired activities. It included: joyful experiences, which facilitated social interaction that made it possible for personal relationships to be transformed. Virtues arose from this transformation, and participants’ experience was that the group engagement supported their well-being. We, therefore, argue that the Dalcroze approach can be used in communities in crisis to facilitate conflict resolution and transform relationships.

## Introduction and background

The purpose of this qualitative hermeneutic phenomenological study is to describe the meaning that members of the Parys community ascribe to their experiences of a Dalcroze-inspired workshop. In this project, members of the Research Niche for the Cultural Dynamics of Water (CuDyWat) at North-West University applied Dalcroze-inspired activities to create a sense of social cohesion among a diverse and randomly selected group of residents and officials of Parys. The workshop enabled them to use music and movement to express their emotions and opinions about their communal water service delivery problems. The research question that guided this inquiry was: What meanings do participants ascribe to their experiences of the Dalcroze-inspired workshop?

The problem is that water resources in South Africa are running dry. There are growing concerns that South Africa, as among the 30 most arid countries in the world, with an average rainfall of less than 600 mm, may not be able to cope with erratic drought conditions, because of anticipated climate change conditions. The latest version of Department of Water and Sanitation (2021) National Water Resources Strategy (Version 3) accentuates the need for local authorities to apply water conservation demand management strategies. All municipal, industrial, and commercial water consumers need to contribute to reducing water loss as far as possible. The country’s municipal water and sanitation infrastructure systems are notorious for leaks and the wastage of costly potable water resources. The DWS now plans to actively promote water use conservation strategies (Department of Water and Sanitation, 2021; Mchunu, 2022).

The town of Parys, which falls under the Ngwathe Local Municipality and operates under the jurisdiction of the Fezile Dabi District Municipality, is frequently subjected to severe water shortages. The town’s water purification infrastructure cannot consistently cope with the inferior quality of raw water at its point of intake on the Vaal River. At the same time, there is an increasing demand for more municipal water due to local population growth. The town’s overworked water and wastewater infrastructure systems are also subject to deterioration. Since the early 2000s, Parys has experienced extensive periods of water-quality issues and infrastructure collapses. In response to a request by a former premier of the Free State Province, the Sasolburg-based company, Sasol Limited, funded a multi-stakeholder consultation research project by the CuDyWat Research Niche at North-West University. The research team focused on the analysis of the impact that the dire water crisis in Parys had on local residents.

Since people use musical experiences to create meaning during difficult times (Bonde, 2011), the research team decided to use a Dalcroze-inspired approach to engage with local stakeholders. The use of Dalcroze-inspired activities in this study is informed by previous research studies (Dutton, 2015; Habron, 2016) that highlight the positive influence of Dalcroze in promoting social transformation within individuals, local communities and the environment. These activities cultivate a desire for self-expression and communication, promoting interpersonal connections and a sense of belonging and well-being (Navarro Wagner, 2016; Sutela et al., 2016). Furthermore, our study draws on the findings from a previous study conducted in the town of Brandfort (Van der Merwe et al., 2019), which demonstrated the significant contribution of a Dalcroze-inspired workshop during a communal water crisis. The findings further revealed that the implementation of the Dalcroze approach effectively fostered a feeling of connectedness and a shared sense of purpose among the participants by making them feel welcome; promoting active engagement; becoming aware of themselves, their space and each other; promoting cooperation and playfulness; experiencing enjoyment; and enabling emotional expression concerning their communal crises.

The findings of these studies motivated the researchers to employ the Dalcroze-inspired approach again, this time in a different context, Parys. This was an ideal opportunity to use Dalcroze-inspired activities as a basis for constructive communication and to interpret the perceptions of municipal water users.

### Music, health, and well-being

The World Health Organization describes health and well-being as a “state of complete physical, mental and social well-being and not merely the absence of disease or infirmity” (Grad, 2002, p. 981). The positive effects of the arts on health and well-being have been studied extensively. Findings have shown that the arts can affect physical and mental health by preventing poor health, promoting good health, and managing and treating various illnesses (Fancourt and Finn, 2019). The growing body of literature on the transformative value and benefits of music for health and well-being illuminates the effective contribution of music participation to the quality of life of a community (Dillon, 2006; Hallam et al., 2012; Lenette and Procopis, 2016). MacDonald et al. (2012) developed a conceptual framework illustrating that the growing multidisciplinary interest in the relationship between music, health, and well-being could enhance musical activities’ social, therapeutic, and communicative benefits. Paton (2011) states that music has a supportive function in enhancing a community’s emotional, mental, physical, and social well-being that goes beyond purely musical entertainment or performance. Participatory music-making serves various social functions in the community, such as involving community members in social communication and activities, and building trust and unity among individuals in the group (Garrido et al., 2016).

Music does not only play a sociocultural role but also has a significant impact on quality of life as it is “an inherently social act and one which contains enormous potential to bring people together and to facilitate various forms of social action” (Murray and Lamont, 2012, p. 76). Music making promotes the community’s wellness by having a calming influence on community members in despair and discomfort; it can be used to inform community members of important health and welfare matters, and music making “can lighten the load of the suffering” (Reigersberg, 2017, p. 134). Small (1998, p. 2) uses the term “musicking” to illustrate that music entails activity and adds that meaning is generated through social and cultural interactions. Stige (2003) views health musicking as an interdisciplinary field that promotes well-being through musical participation. Musical activities in health musicking create meaning in times of hardship and teach strategies to cope with different social problems (Bonde, 2011). A health-musicking perspective supports Dalcroze-inspired activities since the emphasis is on the inter- and intra-personal relationships which develop through musicking to promote health and well-being (Navarro Wagner, 2016). DeNora (2013, p. 1) developed “a grounded theoretical account of how music can be understood to create conditions conducive to wellbeing.” The Dalcroze-inspired workshop for the community members of Parys relates to her theory in so far as the musicking provided momentary respite from distress and afforded participants the opportunity to renew their environments.

### The Dalcroze approach

Émile Jaques-Dalcroze (1865–1950) was a Swiss composer, pianist, and music educator interested in training the body to facilitate inner hearing, musical thinking, learning, and understanding. He was appointed as a professor of solfège^1^ and harmony at the Conservatory of Geneva from 1892 to 1910 (Choksy et al., 1986).

After the First World War, Jaques-Dalcroze was concerned with social reform and cohesion by encouraging students to live in harmony with themselves and those around them. He envisaged the musical expression of human emotion and created an approach that included three branches, namely eurhythmics, solfège, and improvisation; of these, eurhythmics proved to be his unique contribution (Jaques-Dalcroze, 1919). The distinctive Dalcrozian approach to musical learning involves rhythmic bodily movements in response to the elements of music. As a result, scholars develop a bodily awareness awakened by active listening and inner hearing (Le Collège de l’institut Jaques-Dalcroze, 2019). In a Dalcroze class, students explores aspects of time, space and energy through locomotor and non-locomotor body movements.

Movement is the link between the ear and brain, leading the student to an embodied and deeply internalized understanding of music (Le Collège de l’institut Jaques-Dalcroze, 2019). In the Dalcroze approach, the muscular and nervous systems develop simultaneously with the body’s natural rhythms, stimulated by the auditory and visual imaginations of the musical mind. This approach is explained by Juntunen (2004, p. 68) as follows: “Dalcroze Eurhythmics primarily teaches habits of musical action or, more generally, ‘a bodily way of being in sound’, rather than conceptual or abstract knowledge of music.” Dalcroze envisioned making the whole human being more aware, receptive, and imaginative. This approach has spiritual, holistic, and intra-disciplinary dimensions (Habron and Van der Merwe, 2017) and is usually implemented in a group so that learners respond to one another, learn from each other, become aware of the group, and practice mutual respect (Le Collège de l’institut Jaques-Dalcroze, 2019).

In the hands of a caring facilitator, the atmosphere of a Dalcroze class is a safe environment with an active, alert, and reflective approach, and participants learn by “doing” and are encouraged to take risks and develop inter- and intra-personal skills, as well as musical skills (Le Collège de l’institut Jaques-Dalcroze, 2019). In the process, they become more aware of themselves as human beings intersecting in a social ecology where music plays a formidable role in helping them articulate—through movement—how they feel and express what they experience subconsciously. Therefore, Dalcroze-inspired activities were selected as the most appropriate collaborative music-making experience since humans construct meaning from bodily experiences. The research group was particularly interested in understanding the meaning participants ascribed to their experiences of a Dalcroze-inspired workshop. By using music and movement to improve communication between all relevant stakeholders in Parys who wished to engage with the research group on the question of water-related problems, we wanted to find solutions to this critical issue in the community.

In the following sections, we first discuss the research procedures, the Dalcroze-inspired workshop, and the types of activities used. Secondly, in the findings section, we interpret the lived experiences of those who participated in the Dalcroze-inspired workshop and their views on local water-delivery problems. Thirdly we discuss the findings in the context of the relevant scholarly literature.

## Procedures

The best research approach to answer the research question of this study was hermeneutic phenomenology since we wanted to describe the experiences “together with its meanings” of those involved (Hendriksson and Friesen, 2012, p. 1). With this hermeneutic phenomenological inquiry, we intended to describe the experiences “all participants have in common” (Creswell and Poth, 2018, p. 75) during the Dalcroze-inspired workshop.

A one-day workshop was held at Stonehenge on Vaal, a conference venue outside Parys, to ascertain first-hand views and opinions about the water situation from various stakeholders affected by this problem. The research team chose the venue because of its location just outside Parys on the riverbank. Stonehenge has two sizeable halls, where the Dalcroze music and movement session and subsequent discussions were held. The research team met there the day before the workshop to make final preparations for the next day. Invitations to participate in the workshop were randomly distributed to members of the Parys community and to specific key stakeholders identified in previous studies in Parys. The invitations were communicated verbally and via SMS, email, and fax. The verbal invitations were distributed by members of the research team, who went to different areas in Parys to alert stakeholders about the workshop. The municipal management and workers, the Ngwathe Water Forum members, and community members from Tumahole, Schonkenville, and the Parys town area were invited.

The invitation indicated that the stakeholders were invited to participate in a music and movement workshop dealing with water-related challenges facing their community. It also explained that North-West University’s Research Niche for the Cultural Dynamics of Water (CuDyWat) would facilitate the session. Stakeholders were also notified that the transdisciplinary research group was collaborating with Sasol Limited, a partner in a cooperation agreement with the Free State provincial government. It mentioned that the Free State Premier had asked for the assistance of Sasol Limited to investigate the water contamination and shortage challenges in the Ngwathe Municipality. The initial invitations were sent a week before the workshop, up to a day before the event, but did not specify the venue where the workshop would take place. As a precautionary measure to prevent potential disruptions of the event, the venue was only announced 48 h before the workshop.

### Participants

Fifty-three stakeholders from Parys and its neighborhoods and eight researchers participated in the event. The transdisciplinary research team included a biochemist, banker, chemist, political scientist, two musicians, environmental engineer, and two historians. Each one was also encouraged to describe the meaning they ascribed to the Dalcroze-inspired workshop and provide their perspectives on solutions to the water problem. Therefore, there were 62 participants in this study.

Two musicians organized the Dalcroze-inspired workshop in collaboration with the researchers and rehearsed a possible version of the workshop with the researchers the day before. The Parys workshop commenced with a two-hour Dalcroze-inspired session. The purpose of this workshop was to give an opportunity for the expression of emotions about the water challenges through music and movement and to promote creativity, communication, and problem-solving. The Dalcroze-inspired workshop generated a unique opportunity for the diverse participants representing different stakeholder sectors in the community of Parys to interact with each other.

Following the Dalcroze-inspired session, the water meeting focused on participants sharing their encounters regarding the water dilemma. Participants were then asked what action they would take to address the water situation if they were the municipal manager of Parys. The positive emotions generated by the Dalcroze approach broadened their minds (Fredrickson, 2013) and facilitated creative problem-solving. Their suggested solutions were included in an unpublished research report conducted for Sasol Limited on behalf of the office of the Free State Premier. Lastly, the participants were asked what meaning they ascribed to the Dalcroze-inspired workshop. Although some members were initially hesitant when invited to participate in a Dalcroze-inspired movement-to-music session, they were fully prepared to share their deepest feelings with us openly and transparently. The group developed a strong sense of togetherness, and participants could exchange their opinions. This article reports all the findings related to the Dalcroze-inspired workshop.

### Ethics statement

A pre-defined, ethically approved framework guided the research team’s actions. Significant effort ensured an inclusive communication platform between the stakeholders and researchers. Informed consent was obtained from each participant before the workshop to ensure we could use the data for research. Furthermore, pseudonyms were used to ensure participant anonymity and confidentiality. A feedback session with the stakeholders took place six weeks after the workshop to give them an opportunity to give input.

### Data collection

The data-collection process continued during the workshop. While the Dalcroze-inspired workshop was in progress, data were captured of the group in action using photos, videos, sound recordings, and active information gathering by means of notes taken by members of the research team. Data were also collected through focus group interviews and open-ended individual interviews. The objective was to use diverse data collection strategies to glean relevant information on the perceptions and opinions of community members. Immediately after the Dalcroze-inspired workshop, when the focus group interviews were conducted, participants were specifically asked: What meaning do you ascribe to your experiences of the Dalcroze-inspired workshop?

### Data analysis

All data generated during the workshop were qualitatively interpreted as the data were coded, categorized and thematised. The textual data were organized and coded in one heuristic unit in ATLAS.ti, a computer-assisted qualitative data analysis software program (Friese, 2014). ATLAS.ti is a useful tool to organize, manage and support the process of qualitative data analysis. Using the NCT (noticing, collecting, and thinking about things) method (Friese, 2014), data were analyzed and interpreted by moving back and forth between these three iterative steps. Interesting aspects observed in the data were collected and collated in the process of constant comparison (Butler-Kisber, 2018). Throughout the process, we identified patterns and links in the data. This approach to data analysis is illustrated in, among other things, the network views. The data analysis took place in two phases. First, there was descriptive-level open coding (Saldaña, 2013). Later we entered the next conceptual phase in the data-analysis process, and patterns and links were identified.

The themes were subdivided into seven “problem” themes, five “solution” themes, and five “music experience” themes. This article focuses only on the “music experience” themes, namely the meaning participants ascribed to the Dalcroze-inspired workshop and does not elaborate on the water delivery problems and solutions that the stakeholders identified because of the Dalcroze-inspired workshop. The water delivery problems and suggested solutions expressed during the water meetings that followed the Dalcroze-inspired workshop, were included in a report to the Free State Provincial Premier.

### Activities in the Dalcroze-inspired workshop

In a situation where conflict resolution is necessary, the appropriate choice of music and activities is crucial. The workshop aimed to encourage communication between community members by creating an opportunity for them to express their emotions through music and movement. Although the researchers made suggestions for music that they believed could inspire movement, the first author guided the choice of music and activities with reference to her knowledge of the community and its context. As this was mainly an intuitive and spiritual process for the music facilitator, she walked to the music in her living room to get a feel for the music and asked herself: “Will this music make things better?”

The topics of the music were carefully chosen to facilitate reconciliation, such as *Reconciliation Ballet* by Rachel Portman, *You and Your Crown* by Matthew Mole, *Baby can I Hold you* by Tracy Chapman, *Simple Gifts* by Yo-Yo Ma and Alison Krauss, and others. Each activity was built sequentially from the previous activity, and each activity had a social intention, such as making eye contact, being aware of other people, leading and following, synchronizing the beat, making physical contact, celebrating together, giving recognition to the other person, communicating to whom you are going to pass the beat, being expressive and creative together, being sensitive to another person etc. For each activity, clear instructions were given, for example: “Walk with someone you have never met before. Change directions together only by being sensitive to the other person (no talking).” Another example of an instruction for an activity was: “Pair up with someone you do not know. Mirror that person’s movement. Take turns to mirror and be mirrored - take turns to lead and follow.” These are just a few examples.

Our approach relates to Higgins, 2012 third perspective of community music, namely an intentional intervention emphasizing people, participation, diversity and accessibility. We recognized “the value of music to foster intercultural acceptance and understanding” (Higgins, 2012, p. 5). Our choice of musicking was Dalcroze-inspired activities since we have succeeded with this approach in similar community contexts. We acknowledge that other community music approaches that require active engagement in interesting, playful, musical movement activities could have similar findings.

In designing the workshop, the first author was also informed in her choices by the seven types of touch promoted by Greenhead and Habron (2015). She agrees with their statement that “the touch-like nature of sound not only makes contact with the body, inciting physical and emotional movement but also develops awareness of self, others and environment due to the social nature of musical participation in general” (p. 93). They further state that touch activities are suitable to “communicate intentions and feelings to others and receive their responses” (p. 101). Therefore, five of the seven types of touch activities were used as well as one additional category, namely eye contact and imagined contact. Massage and therapeutic touch were not included since sensitive cultural and gender issues were at stake. Recorded music was used since there was no piano available, and the participants related better to more familiar music. The six types of touch/contact included were:

1. Eye contact and imagined contact, greeting with eye contact and throwing an imaginary ball to each other.
2. Direct physical contact with others by clapping each other’s hands.
3. Self-touch: body percussion.
4. Touch and play easy percussion instruments: sound shapes, rattles, bamboo claves and rattles.
5. The touch and manipulation of materials: using balls, ropes, scarves, and other equipment. One group event involved using a large elastic band to connect participants.
6. To touch or be connected using an object such as a rope, scarf or elastic band.

## Findings

The Dalcroze-inspired approach to the water workshop helped people get to know one another, think in a new way about an old problem, put conflict behind them, and, importantly, work on creative solutions. The findings in this article draw on an unpublished research report. In addition, the findings related to the Dalcroze experiences were expanded on, interpreted, and interrogated in the context of the relevant scholarly literature.

### Suffering

**Figure 1:**
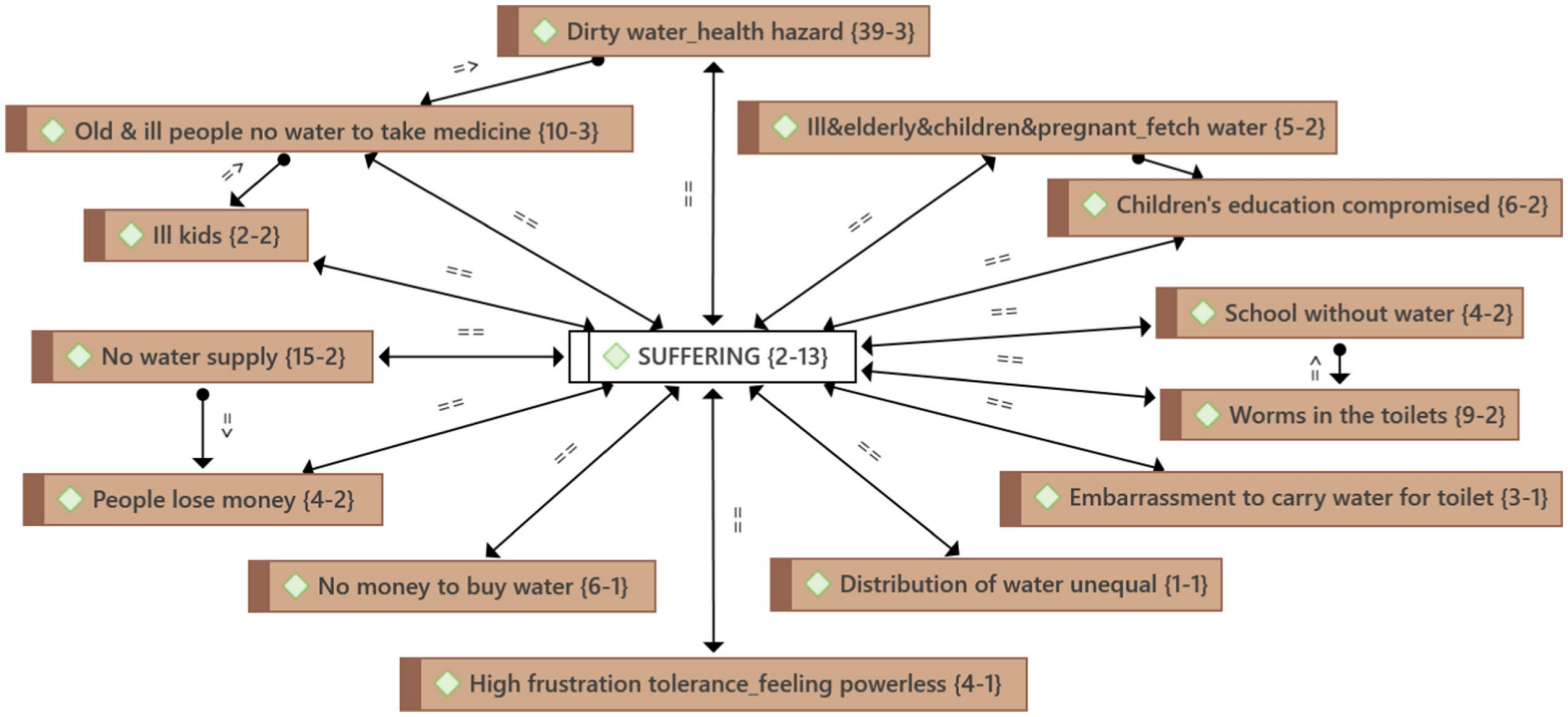
The suffering that the people of Ngwathe endure. The ATLAS.ti networks present the findings visually to illustrate the relationships between codes, categories, and themes. The first number after a code refers to the groundedness, the number of quotes associated with the category. The second number reflects the density, the number of links to other codes (Friese, 2014).

It became clear from the people of Ngwathe’s stories that they suffered tremendously (Figure 1). This suffering is caused by the health risks posed by dirty water. People who do not have money to buy treated water end up drinking the dirty water and become ill. Old and ill people do not even have water to drink with their medicine. In many cases, there is also no water supply, which leads to vulnerable people having to fetch water for themselves. The water problem jeopardizes children’s education. They spend time fetching water when they are supposed to be learning. People also experience embarrassment and frustration with the unfairly and unevenly distributed water. Most participants stated simply and powerfully: “We need water.”

### Emergent themes

**Figure 2:**
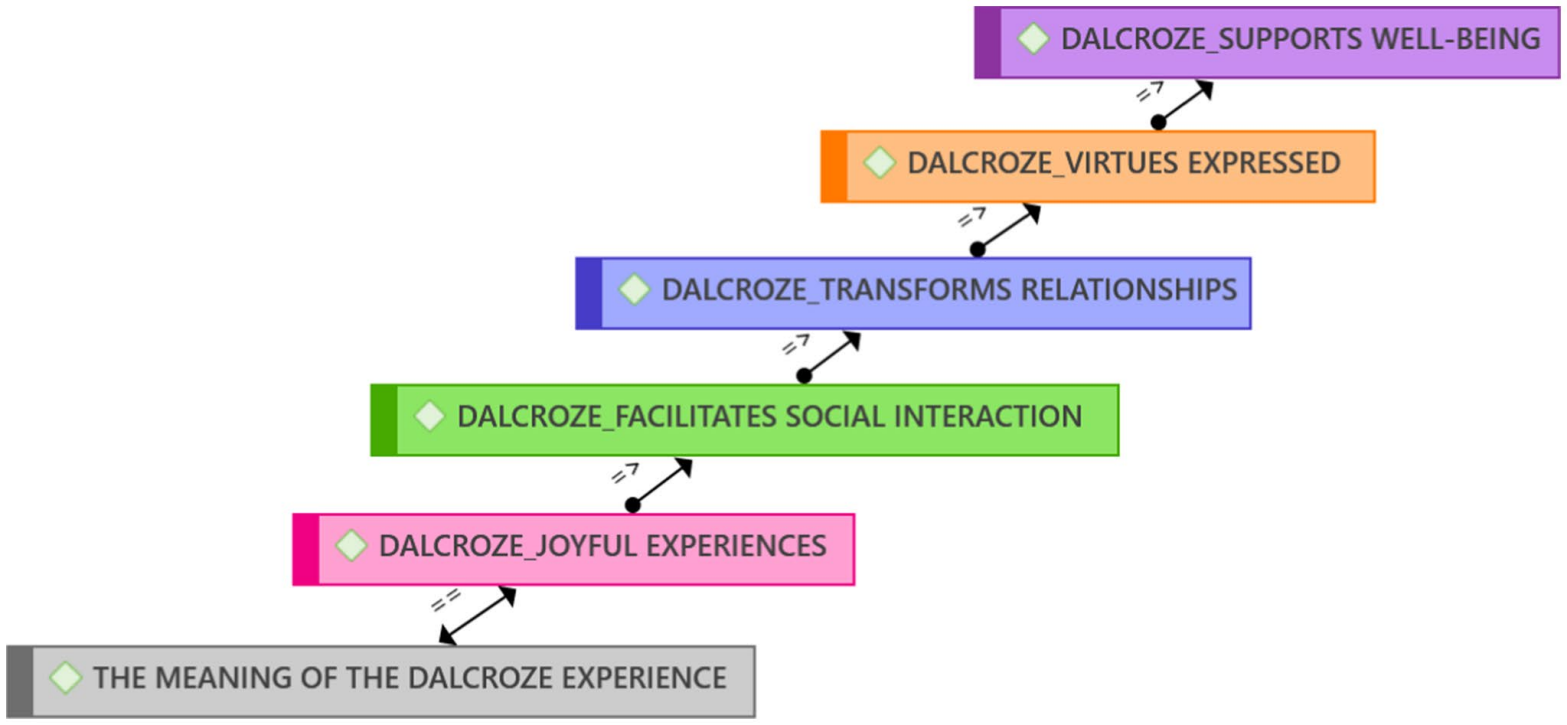
Five emergent themes on the meaning participants ascribed to their experiences of the Dalcroze-inspired workshop.

With the use of the ATLAS.ti qualitative data analysis software, we identified the following five themes from the interviews, focus group interviews, photos, videos, and observations during and after the Dalcroze-inspired workshop (Figure 2):

1. Joyful experiences when participating in Dalcroze activities.
2. Dalcroze activities facilitate social interaction.
3. The Dalcroze approach transforms relationships.
4. Dalcroze-inspired activities foster virtues.
5. Music and movement support well-being.

#### Theme 1: Joyful experiences when participating in Dalcroze activities

**Figure 3:**
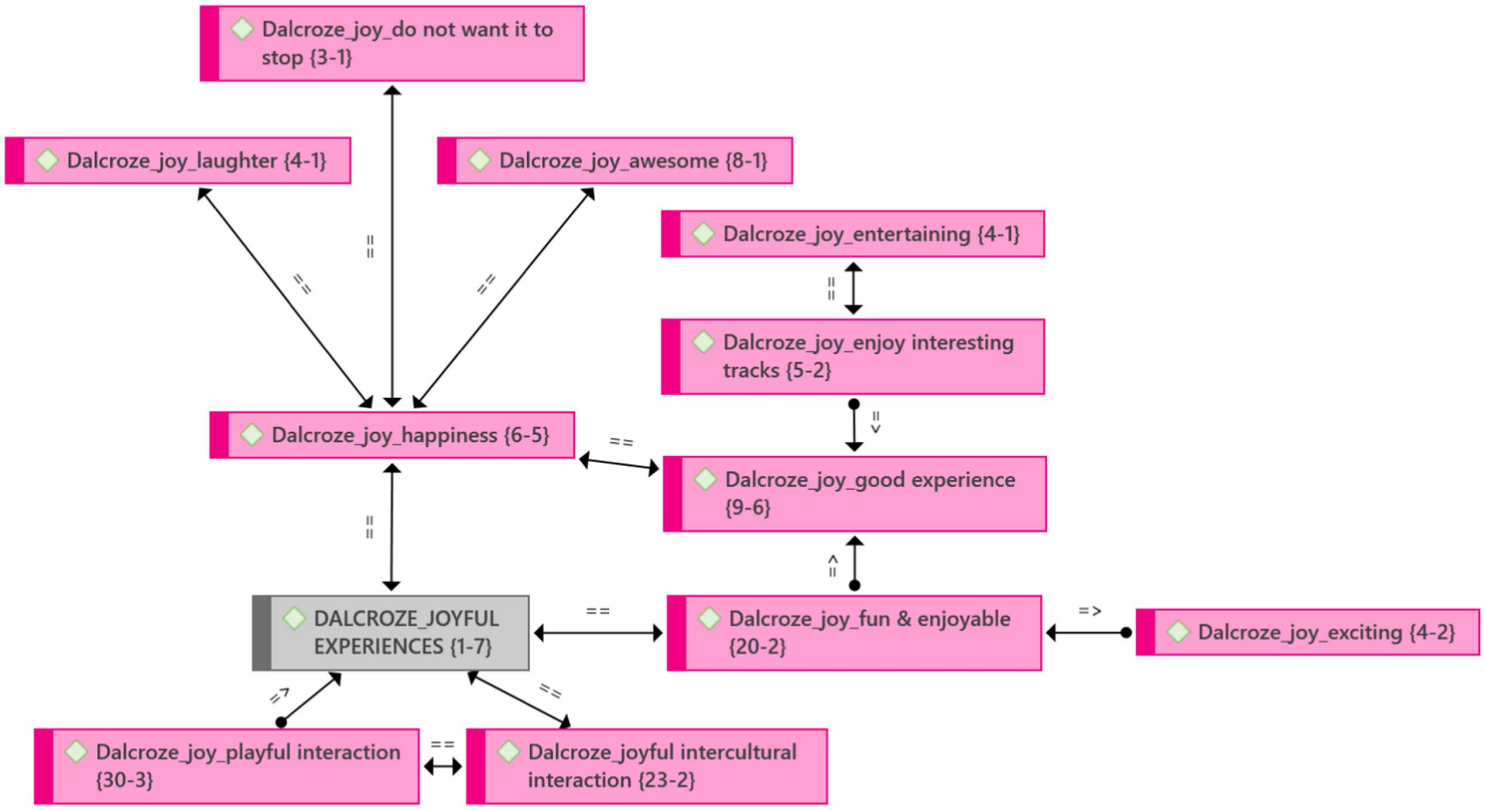
Theme 1: Joyful experiences and emerging categories.

Participants had a joyful experience participating in Dalcroze-inspired activities (Figure 3). They enjoyed it because they found the movement invigorating and exciting. They also enjoyed it because they found the music interesting and the movement activities entertaining. The joy they experienced was expressed as fun, happiness, and a good experience. One of the participants joyfully expressed how the Dalcroze-inspired activities broadened their outlook. The social component of the embodied approach may be the most important reason participants find it enjoyable, which brings us to our next theme, social interaction.

#### Theme 2: Dalcroze activities facilitate social interaction

**Figure 4:**
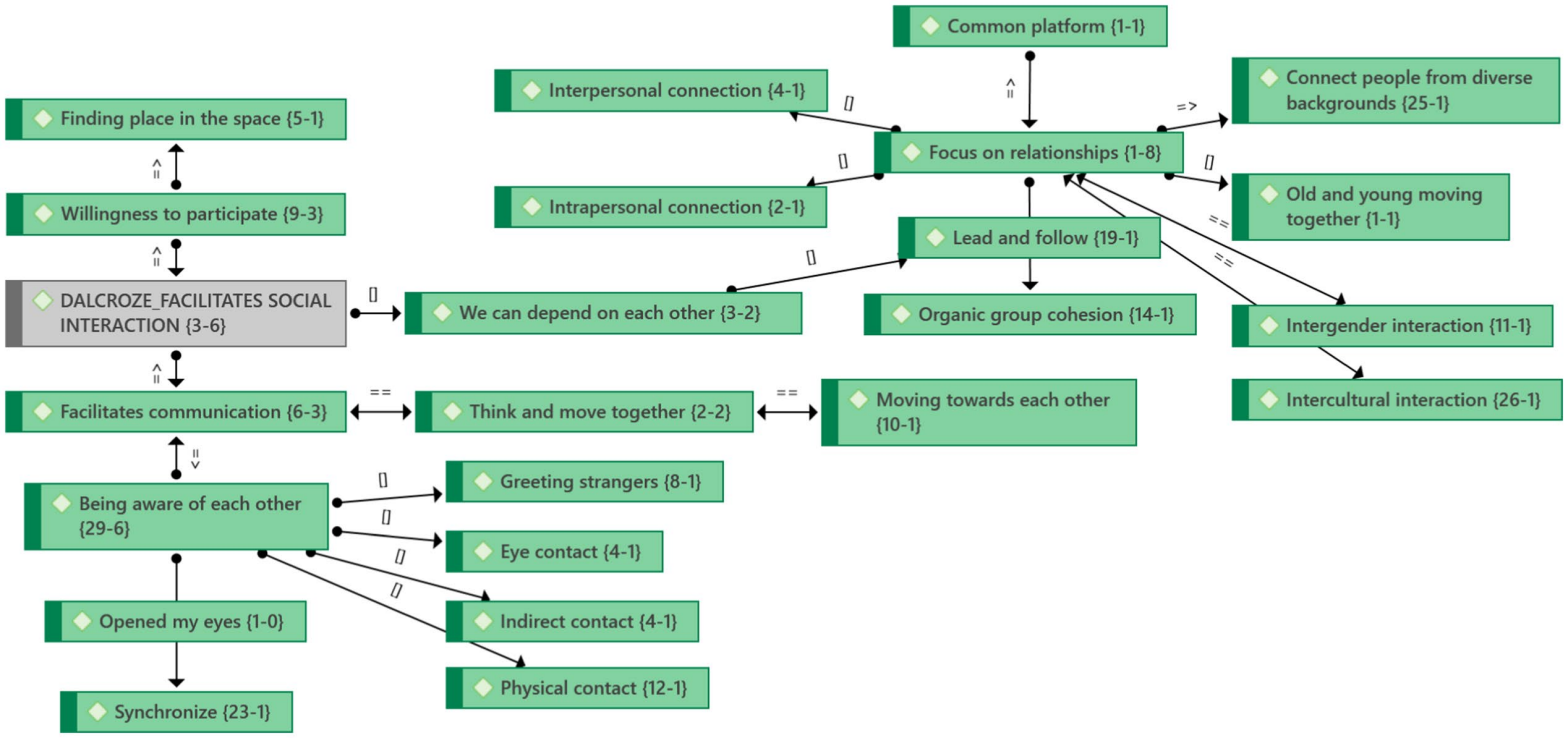
Theme 2: Social interaction and emergent categories.

When people are actively engaged, Dalcroze-inspired activities enable social interaction (Figure 4) because they enable communication. Participants moved in synchrony and could communicate better because of music’s expressive capabilities. One female participant expressed it beautifully:

Yes, and then it leads us nicely into the session, and you know, I think it just oiled the whole meeting. Even the other person said it stopped us from pointing fingers. It helped us to relax and talk from the heart.

Participants explored their roles in the group. They took turns leading and following. This made them realize that they can depend on each other in this workshop and the community when trying to solve the water crises. Their active engagement in the workshop was evidence of their commitment to addressing the water problems of Ngwathe. This social interaction helped relationships to transform.

#### Theme 3: The Dalcroze approach transforms relationships

One of the participants, a representative of the water forum, had instituted a lawsuit against members of the municipality. These members from the municipality were also present at the workshop. The highlight of the meeting was when this participant said: “Uhm, before I went in there, I had three enemies. We are now friends. It was fantastic!”

**Figure 5:**
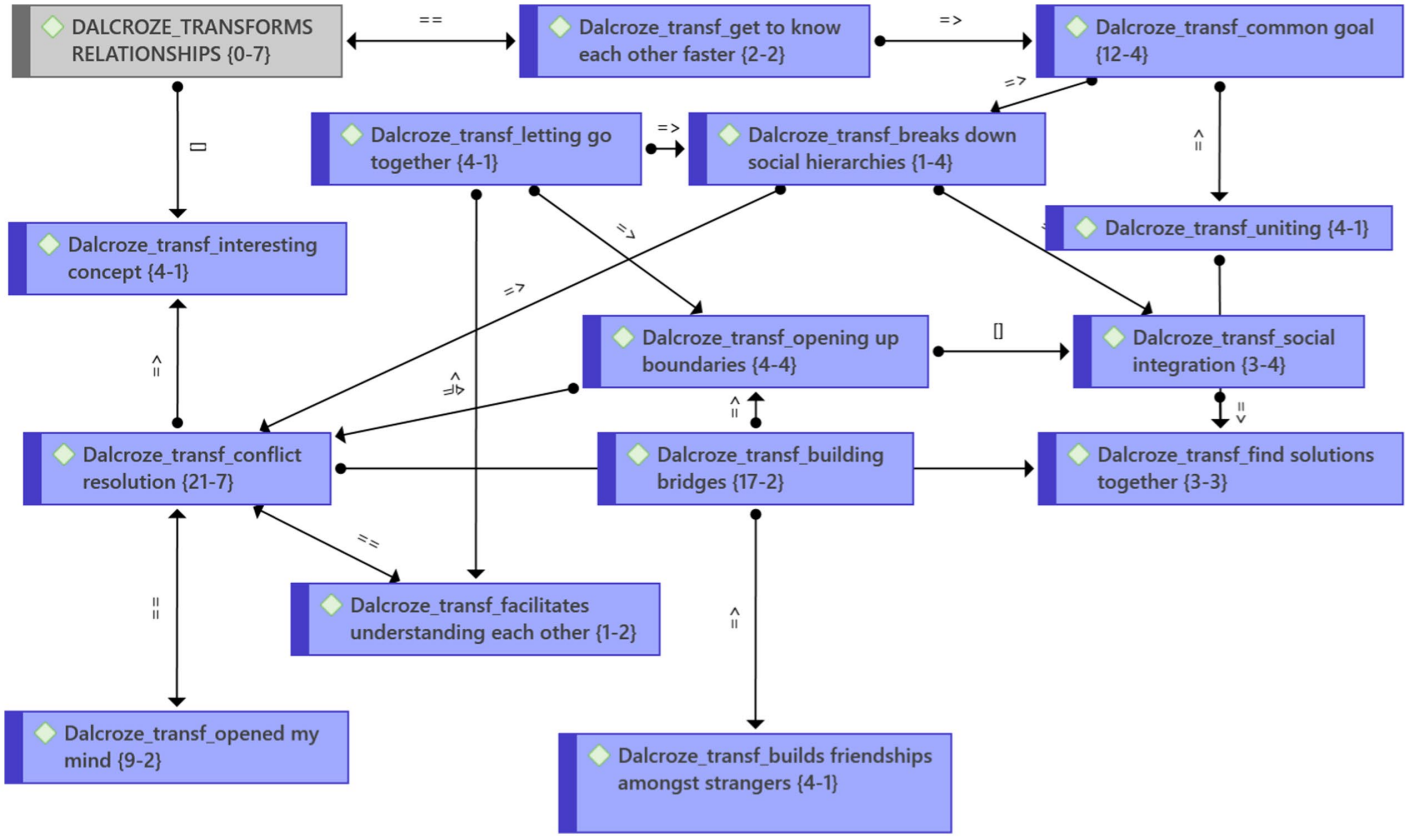
Theme 3: Transformed relationships and emergent categories.

This transformation process (Figure 5) was possible because the music “opens up the boundaries.” Because this was such an interesting new concept for the participants, it opened their minds. Participants got to know each other more quickly. “I’ve never made friends so quickly; it is like quick you meet someone, then you greet them here, then you greet them at another corner, and your mind recognizes, and they also recognize you.” Through the playful interaction, people connected. Music and movement facilitated social integration across ages and cultures, as one participant explained:

… you could even see the different race groups … it was almost like …I do not know … There was no hierarchy, there was no color, there was also no gender. Everybody was trying, following their leader, doing whatever is being done. I was in awe. ... It really broke down those barriers.

Not only does the process help with conflict resolution, but it also helps to build friendships. This transformation process brings out character strengths in people.

#### Theme 4: Dalcroze-inspired activities foster virtues

**Figure 6:**
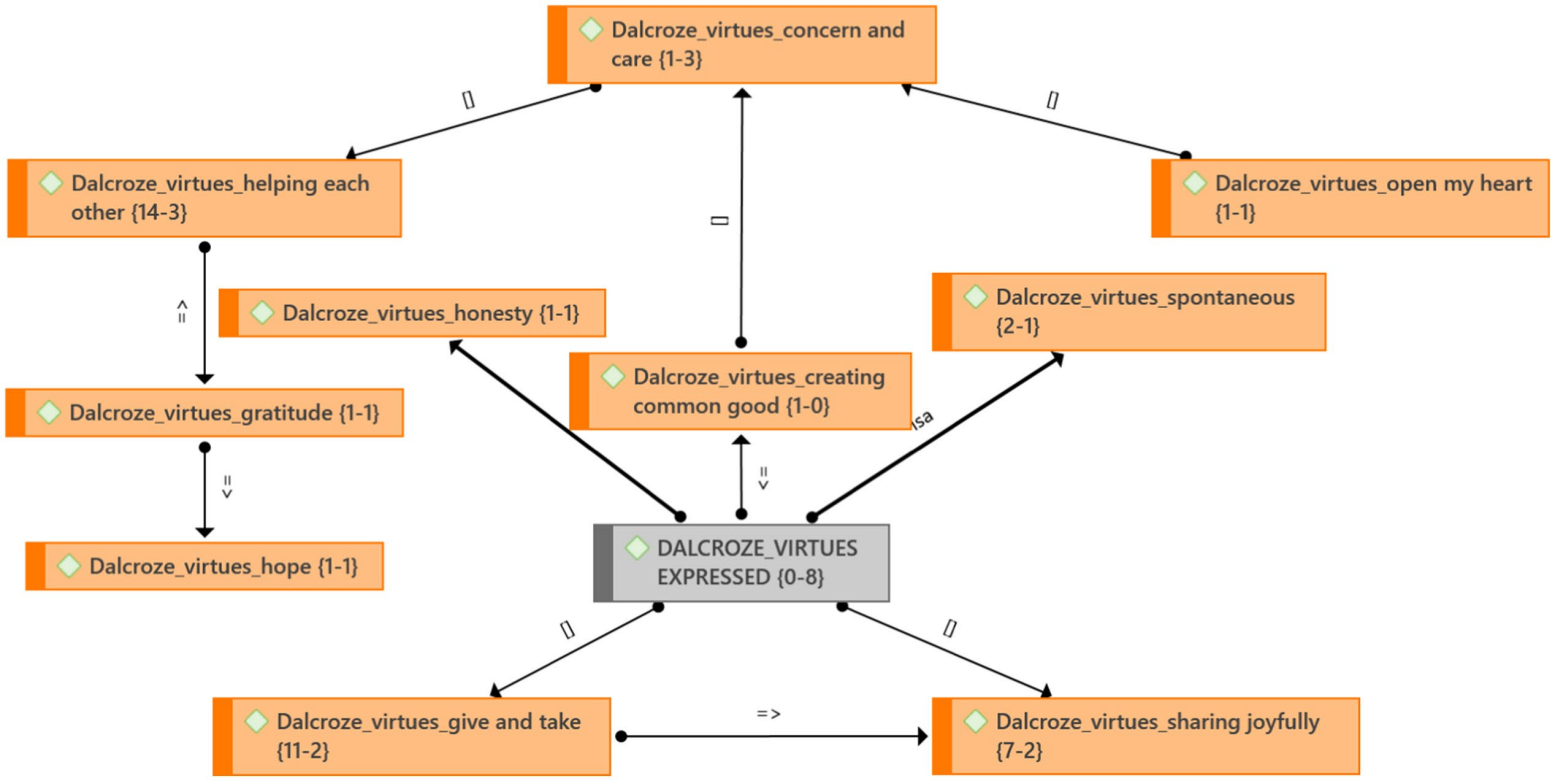
Theme 4: Virtues and emergent categories.

The Dalcroze-inspired activities brought out an uninhibited spontaneity and honesty in participants (Figure 6). “I’m happy. I’m myself. I’m willing to engage, and there is nothing for me to hide from you or anything of that sort.” People felt cared for: “I am very happy there are people concerned about the water problem.” The safe space that was created supported people’s experience of well-being.

The photo and video data showed that participants were helping each other during the Dalcroze-inspired activities. They worked together to create water wave movement improvisations. Furthermore, participants engaged in giving and taking activities. For example, they moved a large elastic band and handed it over to someone else without the elastic snapping. The beat was passed around; scarves were thrown to each other, and balls were rolled to each other. Participants also took turns leading a movement or following someone else’s lead. They gave recognition to each other’s movements in a mirror activity. Recurring quotes after the workshop included “We can depend on each other” and “We should help each other.”

#### Theme 5: Music and movement support well-being

Participants felt welcome at this workshop. Through music and movement, they could express their emotions and sense the music in their bodies. This promoted relaxation and was a source of stress relief. “It was powerful. Music takes the mind far away from stress.”

**Figure 7:**
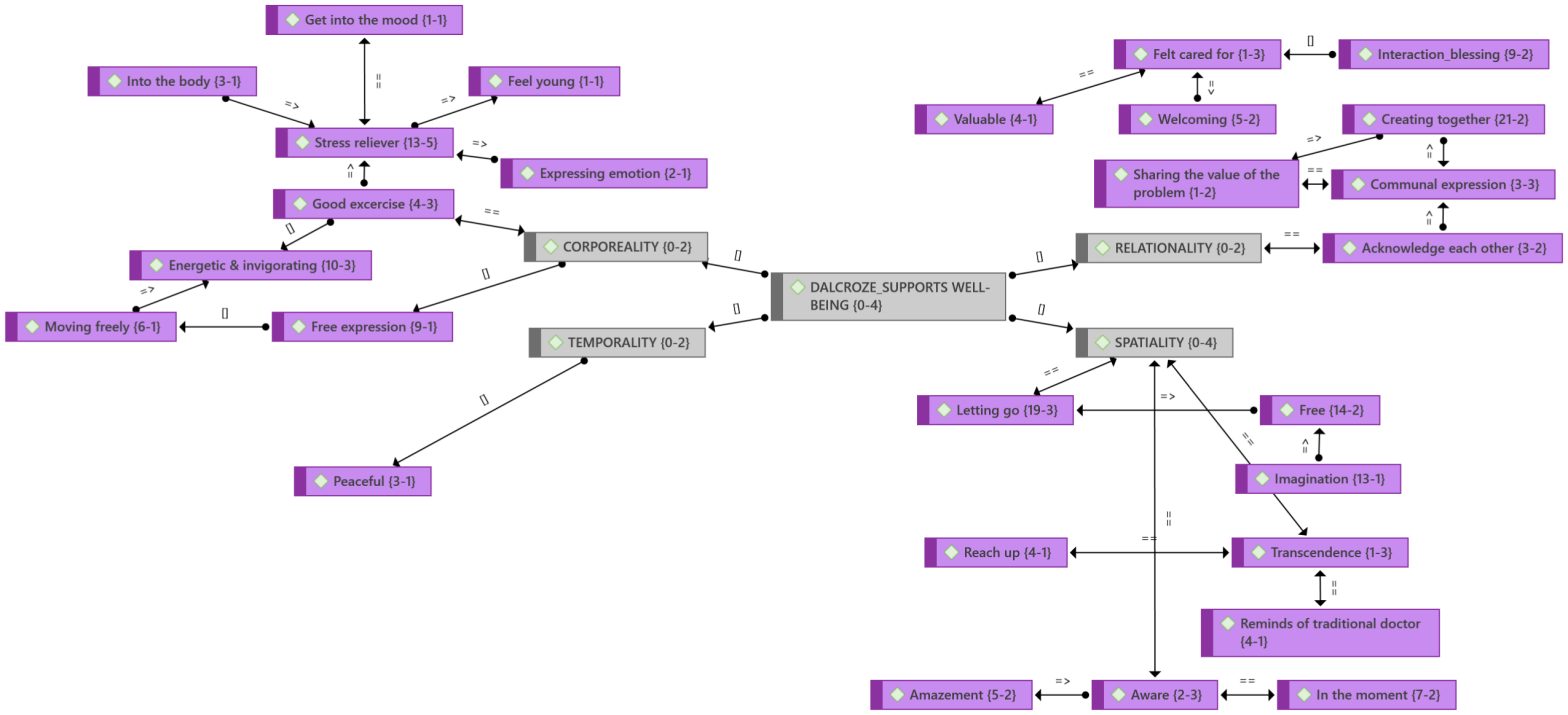
Theme 5: Well-being and emergent categories. The four lifeworld existential of Van Manen (1997, p. 39) form the categories of the well-being theme: lived body (corporeality), lived time (temporality), lived human relation (relationality), and lived space (spatiality). These categories also relate to Van der Merwe and Habron’s (2015) conceptual model of spirituality in music education.

The participants described it as a meaningful experience and a gift. The music helped them to be present in the moment (Figure 7). They were filled with awe and wonder and realized that the Dalcroze approach could help them all. “If we do this on a daily basis, it could help us all. It is better than the six o’clock news on TV.”

#### Water narratives

The closing activity of the workshop required participants to divide into groups to create a movement narrative about their experiences of the water situation in Parys. They were asked to show in movement how they experienced the water situation in Ngwathe. The participants imitated water sounds with percussion instruments, body percussion, and their voices for these improvisations. Some small groups used dramatic storytelling with spoken word, sound effects, and movement to express their experiences. These narratives were devised spontaneously and highlighted the improvisatory nature of the Dalcroze approach. The groups shared the following messages:

- Group 1: How the water purification process at the water plant works and communicated their intention to bring “water to the people.”
- Group 2: The water pressure and quality decrease in the lower-lying areas, and although the municipality provides the water, it does not reach the people, especially not the people in the rural areas.
- Group three 3: The water comes from rain and boreholes, and we need to be proper stewards of the groundwater and surface water.
- Group 4: The impact of leaking pipes.
- Group 5: How the water in the river flows, the beauty of the river and their gratitude for the river.
- Group 6: There is no clear water, and the available water gives them stomach cramps and diarrhea. They pray for water and praise God when it rains.
- Group 7: Tells a story of rain, full reservoirs and an operator that pumps the water to the people, but then the water dries up, and the people are angry at the operator, and they are sad.

## Discussion

We now relate the five themes to the relevant scholarly literature and Jaques-Dalcroze’s own writings.

### Theme 1: Joyful experiences

Elliott (1995) identifies enjoyment as one of the three essential values of music-making as a human pursuit, while Freeman (2002) found heightened enjoyment to be one of the four ways in which students engage in music when a spiritual approach to music education is followed. It is, therefore, a valuable finding of this study that the participants enjoyed the Dalcroze-inspired workshop. This was also a finding in a previous study that the stakeholders of the Brandfort community enjoyed the Dalcroze-inspired workshop experience (Van der Merwe et al., 2019). Jaques-Dalcroze (1912, p. 31) himself said, “I like joy, for it is life. I preach joy, for it alone gives the power of creating useful and lasting work.” Joy is also important because it opens us up for social interaction (Johnson, 2020).

### Theme 2: Social integration

McGuire (2003, p. 15) states, “Collective embodied practices, such as singing or dancing together, can produce an experiential sense of community and connectedness.” In the Brandfort study (Van der Merwe et al., 2019), the researchers found that the Dalcroze-inspired workshop facilitated interaction, cooperation, and connection between people. The current study takes it one step further. Not only did participants interact, but they also experienced synchrony and improved communication as a result. Furthermore, they learnt that they could depend on each other. Ansdell (2014, p. 71) explains that “Musicking allows us to explore, affirm and celebrate our diverse real and symbolic human relationships” and, according to Small (1999, p. 13), “It is in those relationships that the meaning of the act of musicking lies.” Jaques-Dalcroze (1919, p. xii) knew that his approach could help people to express emotions regarding a communal crisis. In 1919, he said, “Now the War is over, the coming generation will experience this need of forming groups for the expression of common emotion.”

### Theme 3: Transforms relationships

Not only can Dalcroze-inspired activities facilitate social integration, but they can also enable the transformation of relationships. Boyce-Tillman (2000, p. 93) states that “Words divide, but sounds unite” and that “When a group of people makes music together, unity is restored.” The Brandfort study (Van der Merwe et al., 2019) revealed that the participants reached a phase of heightened awareness of each other by feeling part of the group. We argue that in the current study, this awareness facilitated the transformation of relationships by opening up boundaries between people. Jaques-Dalcroze (1930a, p. v) says: “I am certain of one thing: that the rightly-directed will can convert mean and selfish instincts into generous and altruistic ones, negative resolves into positive.”

### Theme 4: Virtues expressed

Jaques-Dalcroze (1930b, p. 93) considers art “the outward projection of love and knowledge of beauty and truth.” For him, “receive and give” is the “golden rule of humanity” (1919, p. 63). In the Brandfort study (Van der Merwe et al., 2019), the participants felt positive about being open to each other’s needs and feelings. In the Parys study, openness led to people helping each other. Boyce-Tillman (2007, p. 1418) explains that in a globalizing culture, musical experiences might help us connect to the Other “in ways that are characterized by a combination of respect and empathy.” Virtuous behavior supports our well-being.

### Theme 5: Support well-being

Croom (2015, p. 44) supports the claim that “music practice and participation can positively contribute to one living a flourishing life by positively influencing their emotions, engagement, relationships, meaning, and accomplishment.” The Brandfort study (Van der Merwe et al., 2019) showed that the Dalcroze-inspired workshop positively contributes to participants being able to express their emotions regarding the water crisis, active engagement, and connection with each other. In the Parys study, more explanations were found why Dalcroze-inspired activities supported participants’ well-being: they felt welcome, relaxed, present, and could escape reality. These aspects contribute to participants’ overall well-being. Jaques-Dalcroze (1930c) states that “it cannot be denied that rhythmic movements possess a calming influence upon the nervous system” (p. 159).

## Conclusion

Moving from conflict to open communication and problem-solving is challenging for communities in crises. MacLaren (2009) believes it is more effective to overcome emotional tensions when a conflicting situation is approached positively and meaningfully. She emphasizes that shared bodily experiences are useful for making sense of a mutually conflicting experience, encouraging positive individual transformation.

Similarly, the Brandfort study showed that embodied experiences promoted social interactions and joyful emotions during a communal crisis. The positive findings of the Brandfort study (Van der Merwe et al., 2019) motivated this study’s research undertaking and purpose. Conceptual replication is relevant in qualitative research as its interpretive strengths can lead to numerous discourses on the same phenomenon (Tuval-Mashiach, 2021; Makel et al., 2022). Although the same qualitative approaches were used in both studies, they were applied in different contexts, which supports transferability, adding trustworthiness, dependability, and validity to the research findings (TalkadSukumar and Metoyer, 2019). We developed a distinctive and cumulative body of knowledge, increasing understanding and moving toward a theoretical understanding (see Figure 2) of the phenomenon.

The unique contribution of this study is that the Dalcroze workshop was designed for a community in crisis to facilitate conflict resolution. This article is the first study to show that Dalcroze-inspired activities can facilitate conflict resolution. The Dalcroze-inspired activities encouraged embodied expression and creativity, enabling the participants to develop a new understanding of their association with conflict. The participants recognized the negative effects of conflict situations and identified the need for conflict resolution peacefully to transform relationships.

This study highlighted that joyful, communal, and embodied musical experiences can open participants’ minds to connect, communicate, and creatively solve problems. This joy was affiliated joy (Gabriel et al., 2020); in other words, “joy that was shared with others” (Johnson, 2020, p. 7). The level of arousal during the Dalcroze-inspired workshops was high (Van Cappellen, 2020), generating a lot of energy and urging participants to move. Joy motivates us to reach out to others (Van Cappellen, 2020), as the participants repeatedly mentioned: “We should help each other.” *Joy* sparks an interest in fellow human beings and makes us socially responsive (Izard, 2013). The joyful experience from the communal movement activities was the catalyst for their meaningful experiences.

Meaning is associated with “being a giver” (Dwyer et al., 2017, p. 200), helper, and contributor (Baumeister et al., 2013). Li et al. (2019) explain that meaning leads to “self-transcendent outcomes” (p. 408) when serving something greater than oneself. During this Dalcroze-inspired workshop, participants became aware that they were serving something larger than themselves, namely the community. Meaningful experiences are often associated with a struggle (Dwyer et al., 2017), effort, and worrying (Baumeister et al., 2013) because of a concern for others (Vohs et al., 2019) or an issue, for example, a struggle against injustice (Metz, 2009). Ngwathe stakeholders could share and express their struggle through the Dalcroze-inspired workshop, voice injustices and their needs, and explore possible collaborative solutions. Furthermore, a sense of belonging (Martela and Steger, 2016) and taking care of others (Metz, 2009) enabled the community members to experience the workshop as meaningful. Negative experiences and suffering can lead to transformation and even meaning when the individual can make sense of difficult events (Vohs et al., 2019). Moving together with others and creating water narratives helped participants understand their water crises’ complexities. The Dalcroze-inspired community engagement presented in this article might be transferable to other contexts. In this hermeneutic phenomenological inquiry, the Dalcroze approach was used for a community in crisis to facilitate conflict resolution and transform relationships.

## Data availability statement

The raw data supporting the conclusions of this article will be made available by the authors, without undue reservation.

## Ethics statement

Ethical review and approval was not required for the study involving human participants in accordance with the local legislation and institutional requirements. The participants provided their written informed consent to participate in this study.

## Author contributions

LV facilitated the Dalcroze-inspired workshop, collected and analyzed data, and wrote a large part of the article. DJ wrote the section on Music, health and well-being, and did extensive editing. JT was the leader of the transdisciplinary research team that facilitated the meeting about water, actively collected data in the field and wrote the section on the water, and gave feedback to the various stakeholders. All authors contributed to the article and approved the submitted version.

## Funding

Sasol supported a multi-stakeholder consultation process. Sasol paid for the hire of the venue and the refreshments and branded the venue appropriately on the day of the workshop. Sasol was not involved in the study design, collection, analysis, interpretation of data, the writing of this article or the decision to submit it for publication.

## Conflict of interest

The authors declare that the research was conducted in the absence of any commercial or financial relationships that could be construed as a potential conflict of interest.

## Publisher’s note

All claims expressed in this article are solely those of the authors and do not necessarily represent those of their affiliated organizations, or those of the publisher, the editors and the reviewers. Any product that may be evaluated in this article, or claim that may be made by its manufacturer, is not guaranteed or endorsed by the publisher.

## Footnotes

^1^Solfège is a specific form of aural training, involving the development of sight reading and musical discrimination, typically using solmization syllables.
